# Progress in the Application of Drugs for the Treatment of Multiple Sclerosis

**DOI:** 10.3389/fphar.2021.724718

**Published:** 2021-07-13

**Authors:** Weipeng Wei, Denglei Ma, Lin Li, Lan Zhang

**Affiliations:** ^1^ Department of Pharmacy, Xuanwu Hospital of Capital Medical University, Beijing, China; ^2^ National Clinical Research Center for Geriatric Diseases, Beijing, China; ^3^ Beijing Engineering Research Center for Nervous System Drugs, Beijing, China; ^4^ Beijing Institute for Brain Disorders, Beijing, China; ^5^ Key Laboratory for Neurodegenerative Diseases of Ministry of Education, Beijing, China

**Keywords:** multiple sclerosis, autoimmune diseases, drug therapy, neurodegenerative, neuroprotective

## Abstract

Multiple sclerosis (MS) is an autoimmune and chronic inflammatory demyelinating disease of the central nervous system (CNS), which gives rise to focal lesion in CNS and cause physical disorders. Although environmental factors and susceptibility genes are reported to play a role in the pathogenesis of MS, its etiology still remains unclear. At present, there is no complete cure, but there are drugs that decelerate the progression of MS. Traditional therapies are disease-modifying drugs that control disease severity. MS drugs that are currently marketed mainly aim at the immune system; however, increasing attention is being paid to the development of new treatment strategies targeting the CNS. Further, the number of neuroprotective drugs is presently undergoing clinical trials and may prove useful for the improvement of neuronal function and survival. In this review, we have summarized the recent application of drugs used in MS treatment, mainly introducing new drugs with immunomodulatory, neuroprotective, or regenerative properties and their possible treatment strategies for MS. Additionally, we have presented Food and Drug Administration-approved MS treatment drugs and their administration methods, mechanisms of action, safety, and effectiveness, thereby evaluating their treatment efficacy.

## Introduction

Multiple sclerosis (MS) is one of the malignant diseases that threaten the health of teenagers. Changes in environment and daily habits modulate the occurrence rate of this disease. MS is a chronic autoimmune disease of the central nervous system (CNS), which is characterized by demyelination and loss of nerve axons induced by an abnormal CNS-directed immune response and inflammation (Dendrou et al., 2015).

MS exhibits different phenotypes. In most patients, it is characterized by recurring clinical symptoms followed by complete or partial recovery, that is, typical relapsing-remitting MS (RRMS). After a period, the nervous system gradually deteriorates and a stage termed secondary progressive MS (SPMS) is established. However, some patients have accumulated disabilities caused by endless progression of the disease from the onset, which leads to primary progressive MS (PPMS) and clinically rare progressive relapsing MS (PRMS) (Gajofatto and Benedetti, 2015; Correale et al., 2017; De Angelis et al., 2018). The course of MS varies greatly among patients. Although significant progress has been made in the treatment in recent years, MS remains one of the most common causes of neurological dysfunction in young people. It mainly affects young and middle-aged individuals, with approximately 30 yr as the peak age of onset, and the ratio of male to female patients is approximately 1:2. To date, the etiology and pathogenesis of MS have not been fully elucidated. Long onset time, multiple lesions, and wide spread are the clinical characteristics of MS, disseminating in time and space and greatly influencing function, economy, and quality of life. The cost of MS treatment is quite high and increases with an increase in disability. While current treatment options with different immunomodulatory or immunosuppressive effects mainly reduce the frequency and severity of recurrence, they cannot cure the disease (Klotz et al., 2019).

In recent years, based on the efforts of researchers and the study of MS drug therapy, new therapeutic drugs have been discovered and developed. MS is a complex disease and is classified as an organ-specific T cell-mediated autoimmune disease, its pathogenesis is not yet fully understood. Although several proven genetic elements have been described with regard to MS, many several environmental risk factors are shown to play an important role with the focus on vitamin D or ultraviolet B exposure, Epstein-Barr virus infection, obesity, or smoking (Dobson and Giovannoni, 2019; Teymoori-Rad et al., 2021). With increasing understanding of the pathogenesis of MS, it has been elucidated that environment factors, rather than genetic factors, play an important role in susceptibility. Furthermore, it is known that innate and adaptive immune systems and their effector cells, such as microglia, activated macrophages, and B and T lymphocytes, can influence the pathogenesis of MS (Oh et al., 2018; Yamout and Alroughani, 2018). This discovery not only revealed a new therapeutic target but also laid a foundation for the search of new therapeutic drugs. In this review, we aimed to summarize and discuss new findings in MS drug therapy, including drugs currently undergoing trials and those already approved by the Food and Drug Administration (FDA), focusing on latest reports and progress in drug treatment of diseases to provide a reference for further elucidating the pathogenesis and potential therapeutic targets of MS.

### Types and Characteristics of MS

MS is the most common inflammatory disease of the CNS in young people. Its clinical symptoms vary, including sensory and visual impairment, limb weakness, tremor, limb movement disorder, visceral dysfunction, and mental depression. Lesions in MS correspond to local demyelination and inflammation, leading to glial reactions and eventual axonal injury. These lesions scatter throughout the CNS, including white and gray matter (Myhr, 2008; Bigaut et al., 2019). Traditionally, according to the characteristics of the clinical course, MS can be divided into four different clinical phenotypes: RRMS, SPMS, PPMS, and PRMS. According to the incidence and prognosis of MS, there are two rare clinical types of the disease, which overlap with the above clinical types, including benign and malignant MS. In benign MS, the number of relapses reduces within 10–15 yr after onset, while the nervous system still functions well. In contrast, patients with malignant MS exhibit sudden onset of the disease, which progresses rapidly with subsequent neurological deterioration, resulting in disability or death.

### Relapsing Remitting Multiple Sclerosis, RRMS

RRMS has a remission cycle of relapse and remission, which is characterized by acute remission (relapse) and relatively stable intermission (remission). In this phenotype, a patient recovers after each attack, leaving no or only mild sequelae. The condition of the two relapse intervals is stable and has the best response to treatment, and up to half of the patients with RRMS may exhibit the secondary progressive type of MS after a period. The incidence rate of RRMS among women is approximately twice that among men, and approximately 85–90% of patients with MS present this phenotype. Its pathogenesis is the production of lumpy demyelinating areas of varying sizes in the neurocellulose area, and its pathological characteristics are varying degrees of inflammatory cell infiltration, demyelination, axonal injury, and astrocyte hyperplasia. RRMS is the most common type of MS and the hot spot of clinical research.

### Secondary Progressive Multiple Sclerosis, SPMS

After 10–15 years of illness, approximately 50% of RRMS patients no longer experience relapse and remission, and show slow progressive aggravation, which manifests as a stage of continuous deterioration of disability with or without seizures.

### Primary Progressive Multiple Sclerosis, PPMS

PPMS is rare and onset occurs in a relatively older age, accounting for approximately 10% of MS occurrence. The disease has a course duration of 1 yr or more; it progresses slowly showing only short-term, insignificant symptom improvement with no remission or recurrence and exhibits poor response to treatment.

### Progressive Relapsing Multiple Sclerosis, PRMS

PRMS is clinically rare and is characterized by gradual development and aggravation, occasional recurrence, and continuous progression between two relapses.

### Application of Drugs for the Treatment of MS

#### Drugs Approved by FDA

The treatment of MS is mainly based on the use of immunosuppressants and immunomodulators. Until 1993, MS treatment was not licensed; however, several treatments are now available (Ziemssen, 2011; Antonio Garcia Merino, 2014; Boster et al., 2017; Kidd et al., 2017). Currently, approved drugs for the treatment of MS are usually disease modifiers, which only reduce the incidence of the disease and delay its progression in some patients. It is believed that these treatments are only effective against the inflammatory component of the disease (Bagherpour et al., 2018). With rapid progress in the development of effective MS therapeutic drugs, a variety of these drugs are now marketed. Although many drugs are used to treat MS in the clinic, only a few of them have been approved by the FDA. At present, the products approved by the FDA for the treatment of MS include interferon (IFN)-β, glatiramer acetate (GA), teriflunomide, fingolimod (FTY), mitoxantrone, natalizumab, dimethyl fumarate, and alemtuzumab (Table 1).

**TABLE 1:** List of drugs approved by the FDA for the treatment of MS.

Product name	Dosage and Administration	Pharmacological actions and Mechanisms	Adverse reactions	Approved
IFN-β-1b (Betaseron) Yu et al. (2015)	250 µg i.H. every 2 days	Activates the JAK/STAT pathway connected by IFN receptor, resulting in transcriptional changes in immune and anti-proliferative genes, and reduces the migration of lymphocytes across the BBB	Influenza-like syndrome, skin reaction at injection site, headache, leukopenia, etc.	1993
IFN-β-1a (Avonex) Pavelek et al. (2020)	30 µg i.m. once a week	Inhibits the proliferation of MBP-specific T cells and their penetrating migration to the BBB, reduces the production of pro-inflammatory factors, and induces the increase of anti-inflammatory factors	Influenza-like syndrome, anemia, fever, myalgia, weakness, etc.	1994
Glatiramer acetate Song et al. (2020)	20 mg i.H. once a day	Competitively binds MHC Ⅰ and Ⅱ molecules of APC to block MBP specific T cell receptor, inhibits T cell proliferation, down-regulates the secretion of inflammatory cytokines, and up-regulates the production of brain-derived neurotrophic factor	Skin reaction at the injection site, palpitations, dyspnea, chest pain, vasodilation, etc.	1996
Mitoxantrone Edan et al. (2004); Burns et al. (2012)	4–12 mg i.v.gtt every 3 mo	Embeds into DNA base molecules to inhibit DNA synthesis, inhibits the presentation of antigens for T and B cells, reduces the secretion of proinflammatory cytokines, such as TNF-α, and enhances anti-inflammatory response	Intestinal reactions, alopecia, peripheral blood leukopenia, abnormal liver function, etc.	2000
IFN-β-1a (Rebif) Hupperts et al. (2019)	44 µg i.H. every 3 wk	Promotes the balance of Th1 and Th2 cells, reduces the secretion of proinflammatory cytokines, enhances the expression of inhibitory cytokines, and reduces the entry of T cells into the CNS through the BBB	Influenza-like syndrome, skin reaction at the injection site, myalgia, abdominal pain, elevated liver enzymes, etc.	2003
Natalizumab Zhovtis Ryerson et al. (2020)	300 mg i.v. every 4 wk	Anti-α4 integrin monoclonal antibody; binds and blocks the interaction between α4 integrin and ligand and prevents lymphocytes from entering the CNS through the BBB	Headache, urinary tract infections, abdominal pain, fatigue, joint pain, gastroenteritis, etc.	2004
Fingolimod Imeri et al. (2021)	500 µg p.o. once a day	S1P receptor modulator; protects and repairs neurons through the BBB and prevents central memory T cell subsets from migrating to the CNS	Systemic virus infection, headache, influenza, gastrointestinal discomfort, abnormal liver function, angina pectoris	2010
Teriflunomide Buron et al. (2021)	7 or 14 mg p.o. once a day	Dihydroorotate dehydrogenase inhibitor; reduces DNA synthesis, inhibits T and B cell proliferation and production of cytokines, and inhibits intercellular adhesion molecule production	Dyspnea, renal failure, hypertension, leukopenia, alopecia, etc.	2012
Tecfidera Naismith et al. (2020a); Naismith et al. (2020b)	240 mg p.o. twice a day	Regulates the levels of Nrf2 and glutathione in T cells, activates antioxidant genes, and promotes the transformation of Th1 to Th2	Abdominal pain, diarrhea, nausea, skin itching, rash, erythema, etc.	2013
Alemtuzumab Gross et al. (2016); Paterka et al. (2016)	12 mg i.v. once a day	CD52 monoclonal antibody; induces the clearance of T and B cells and increases the secretion of brain-derived neurotrophic factor	Rash, headache, fever, other autoimmune diseases, etc.	2014
Peginterferon beta-1a Menge et al. (2021)	125 µg I.H. every 2 wk	Reduces the expression of adhesion molecules on the surface of T cells, inhibits the activation of T cells, and reduces the infiltration of the CNS	Influenza-like symptoms, injection site reaction, and deterioration of depression	2014
Daclizumab Cohan (2016); Gold et al. (2016)	150 mg i.H. once a month	CD25 monoclonal antibody; inhibits IL-2 receptor signal transduction and T cell activation and proliferation	Severe infections and skin reactions, abnormal liver function, etc.	2016
Ocrelizumab Patel et al. (2021)	300 mg i.v. every 2 wk	Monoclonal antibodies against CD20 on immature and mature B cells; removes CD20 positive B cells using CDC and ADCC	Skin reaction at the injection site, headache, malignant tumor, etc.	2017
Cladribine Miravalle et al. (2021)	10 mg p.o. (3.5 mg/kg cumulative dose over 2 yr)	Nucleoside analogue; inhibits DNA synthesis and DNA chain termination and cytotoxic to lymphocytes and monocytes	Respiratory tract infection, headache, lymphocytopenia, etc.	2019
Siponimod Spampinato et al. (2021)	250 μg or 2 mg p.o. once a day	S1P-1 receptor modulator; enters the brain and CNS of MS patients through the BBB, binds to S1P receptor, promotes myelin regeneration, prevents activation of harmful cells, delays disability progression, and preserves cognitive function	Increased blood pressure, decreased heart rate, delayed atrioventricular conduction, macular edema, respiratory and skin infections, etc.	2019
Ozanimod Lamb (2020); US Food and Drug Administration (2020)	250 μg p.o. once a day	A novel S1P and dual subtypes of S1P1 and S1P5 receptor modulators; enters the brain and CNS through the BBB and binds to S1P receptors to promote myelin regeneration, prevents activation of harmful cells, delays disability progression, and preserves cognitive function in patients	Respiratory tract infection, urinary tract infection, transient decrease of heart rate and delayed atrioventricular conduction, elevated blood pressure, etc.	2020

i.H., subcutaneous injection; i.m., intramuscular injection; i.v.gtt, intravenous drop infusion; i.v., intravenous injection; p.o., per os; IFN, interferon; BBB, blood-brain barrier; MBP, myelin-basic protein; MHC, major histocompatibility complex; APC, antigen-presenting cells; TNF, tumor necrosis factor; Th, helper T cells; CNS, central nervous system; S1P, sphingosine-1-phosphate; Nrf2, nuclear factor erythroid 2-related factor 2; IL, interleukin; CD, cluster of differentiation; CDC, complement-dependent cytotoxicity; ADCC, antibody dependent cellular cytotoxicity.

### Interferon (IFN)

IFN was the first cytokine discovered and studied in humans. It can activate macrophages, increase natural killer cell activity, and inhibit virus replication; it was originally used in antiviral therapy. IFN can be divided into three types according to their origin and structure: α, β, and γ. IFN-β is effective, IFN-α is ineffective, and IFN-γ can aggravate the disease (Wittling et al., 2020; Shen et al., 2021). IFN-β has been recommended as a first-line drug for patients with RRMS by the FDA. The mechanism of action of IFN-β involves the inhibition of lymphocyte proliferation and antigen expression, regulation of anti-inflammatory phenotypic cytokinesis products in the circulatory system and CNS, inhibition of T cell matrix metalloproteinase activity, and reduction of inflammatory T cell migration (Shahi et al., 2020).

The first generation of IFN-β was approved by the FDA in 1993 and is the earliest disease-modifying treatment used for MS. Two kinds of IFN-β compounds exist, IFN-β-1a and IFN-β-1b, both of which must be injected (Zettl et al., 2018). IFN is a small protein that can be degraded or cleared quickly; thus, it is administered frequently, ranging from every other day to once a week (Jain and Jain, 2008). Currently, three parenteral IFN-β preparations are approved for the treatment of MS: IFN-β-1b is subcutaneously injected every other day, IFN-β-1a is subcutaneously injected three times a week, and IFN-β-1a is injected intramuscularly once a week. IFN-β is an immunomodulatory drug with multiple targets; however, its exact mode of action is not yet completely clear (Filippini et al., 2017). The therapeutic effect of IFN-β has been determined, and its greatest advantage is that it has no deleterious side effects, such as malignant tumors or teratogenicity (Rommer and Zettl, 2018). Its limitations include side effects, such as skin reactions (from erythema and itching to infection and even necrosis), influenza symptoms, muscle pain, joint pain, chills, headache, and body weakness. Therefore, injection-related adverse events can negatively influence compliance, and the need for frequent administration may become an obstacle to MS treatment (Mohr et al., 2001; Patti, 2010; Beer et al., 2011; Menzin et al., 2013).

### Glatiramer Acetate (GA)

GA, approved by the FDA in 1996, is an immunomodulating amino acid copolymer (Aharoni, 2013). Its mechanism of action involves the activation of T cells and induction of Th2 cell production. Th2 cells can promote the production of anti-inflammatory cytokines, such as interleukin (IL)-4, IL-10, and TGF-β, thus playing an immunomodulatory role. Commonly, a dose of 20 mg once a day is administered subcutaneously. At present, no additional benefit of a higher GA dose has been found, and the effect of the drug on the recurrence rate of MS after 2 yr of treatment is similar to that of IFN-β (Comi et al., 2011; La Mantia et al., 2015; van Dijkman et al., 2018). Although GA is safe, some patients still experience adverse reactions. Skin reaction at the site of injection is frequent, and fat atrophy may occur. Injection-related reactions include blushing, chest pain, palpitations, urticaria, and dyspnea; these side effects and the need for daily injection lead to a huge burden, which negatively influences treatment sustainability (Patti, 2010; Beer et al., 2011; Krysko et al., 2020).

### Teriflunomide

Teriflunomide is an inhibitor of pyrimidine synthase (dihydroorotate dehydrogenase). It plays a neuroprotective role by inhibiting dihydroorotate dehydrogenase, blocking the synthesis of DNA and RNA, and reducing the proliferation of immune cells (Bar-Or et al., 2014). Teriflunomide is a daily oral disease modification therapy approved for the treatment or relief of recurrent MS. This compound is the main active metabolite of leflunomide, a drug approved for the treatment of rheumatoid arthritis. Teriflunomide selectively and reversibly inhibits dihydrophosphate dehydrogenase, which is the key mitochondrial enzyme for deoxypyrimidine synthesis required for the rapid division of B and T lymphocytes. Through this cellular static effect, teriflunomide has the potential to limit the immune response that leads to MS activity (Gold and Wolinsky, 2011; Confavreux et al., 2014). Related studies have shown that 14 mg of the drug can significantly reduce the annual recurrence rate of MS per patient and the risk of disability progression lasting at least 12 wk. Further, it has been reported that 7 mg of teriflunomide can significantly reduce the annual recurrence rate of MS; however, this dose has no significant effect on disability progression. In addition, extended studies have shown that long-term (approximately 8.5 yr) treatment with teriflunomide can maintain the efficacy of drug. Diarrhea, nausea, thinning of hair (alopecia), and increased concentration of alanine aminotransferase are the most common adverse reactions associated with teriflunomide (O'Connor et al., 2011; Confavreux et al., 2012; Wolinsky et al., 2013; Confavreux et al., 2014).

### Fingolimod (FTY)

FTY, the first oral immunosuppressant and a sphingosine-1-phosphate receptor (S1PR) modulator, was approved by the FDA in 2010 as an oral drug for the treatment of MS. The main mechanism of action is to inhibit the release of lymphocytes from the peripheral lymphoid tissue by binding to S1PR on the surface of lymphocytes after phosphorylation or to induce lymphocytes in peripheral blood to migrate back to the peripheral lymphoid tissue and reduce their entry into the CNS. In addition, FTY can directly regulate the expression of S1PR on the surface of oligodendrocytes and neurons through the blood–brain barrier (BBB), and plays a role in neuroprotection and repair. Five different types of S1PRs exist, among which FTY binds to S1PR 1, 3, 4, and 5, and the immunomodulatory effect of FTY may be mediated by S1PR1. The internalization of the receptor makes it impossible for immune cells to leave the lymphoid tissue or enter the CNS to promote an autoimmune response. The results of clinical trials show that FTY is effective in patients with recurrent MS, and oral administration can reduce the treatment burden of the injection (Cohen et al., 2016; Matko et al., 2020). The related side effects of FTY in the trial included cardiac autonomic nervous dysfunction, high infection rates (especially herpes infection), melanoma, and eye problems associated with the development of macular edema. Additionally, animal studies have reported teratogenicity and embryonic lethality, including organ defects, especially permanent truncus arteriosus and ventricular septal defects (Cohen et al., 2010; Findling et al., 2020).

### Mitoxantrone

Mitoxantrone, an immunosuppressant, is a topoisomerase II inhibitor that inhibits cellular DNA replication, transcription, and repair. It was originally used to treat diseases, such as myeloid leukemia and prostate cancer. In 2000, mitoxantrone was approved by the FDA for the treatment of patients with worsening RRMS and SPMS, and its effect on the treatment of RRMS was definite. Mitoxantrone is similar to an embedding agent in the treatment of MS when it is embedded in DNA base molecules, which inhibits DNA synthesis and the presentation of antigens, such as T and B cells, reduces the secretion of inflammatory cytokines, such as tumor necrosis factor (TNF), and plays an immunosuppressive and neuroprotective role (Jeffery and Herndon, 2004; Martinelli et al., 2009). The drug is administered once every 3 mo for 2 yr, which can reduce the recurrence frequency, lesion formation, and disability rate associated with MS. However, the clinical application of mitoxantrone is limited because of its side effects, including cardiotoxicity, hair loss, constipation, and abnormal liver function.

### Potential New Drugs for MS

MS is a complex inflammatory autoimmune disease of unknown etiology. It is believed that the pathogenesis of MS mainly occurs as an immune response to myelin or myelin-forming cells (oligodendrocytes) owing to the presence of abnormally activated T cells in the CNS, which leads to progressive demyelination in the CNS and neurodegenerative diseases. Macrophages, self-reactive CD8^+^T cells, Th1 and Th17 cells, and clonal expanded B cells have been reported to dominate the inflammatory infiltration of the BBB. Further, a number of autoantibodies and autoreactive T cells have been found in patients with MS (Bittner et al., 2014; Lombardo et al., 2019). Inflammatory cytokines produced by self-reactive T cells passing through the BBB and the CNS can cause damage to myelin and surrounding tissues, and microglia and astrocytes in the CNS are activated during inflammation and produce pro-inflammatory mediators that worsen the disease (Dendrou et al., 2015). The classification of MS is also an important reference for the choice of therapeutic drugs. It is suggested that we should combine the typing of MS with the factors affecting the CNS and the corresponding types of immune cells to study whether different influencing factors act on different parts of the nervous system, resulting in different types of MS in order to further study the pathogenesis of MS and find a new target for MS therapy. Therefore, based on the above, we focus on adaptive immune responses in T and B cells, as well as myeloid cells of the innate immune system (dendritic cells (DCs), astrocytes, microglia, and oligodendrocytes), to discuss the role of drugs in the treatment of patients with MS and some animal models with the aim of finding new targets and strategies for the development of therapeutic drugs for MS.

Although several therapeutic drugs for MS are available in the clinic, immunomodulatory drugs to control the recurrence of MS or completely cure the disease are not enough and the treatment cost is quite high. However, because of deleterious side effects, adverse reactions caused by cyclophosphamide in the treatment of MS cannot be ignored; for example, cyclophosphamide in the treatment of MS often causes adverse reactions, such as peripheral leukopenia, gastrointestinal reactions, hemorrhagic cystitis, malignant tumors, and increased infertility. Thus, it is not used clinically (La Mantia et al., 2007; Patti and Lo Fermo, 2011; Findling and Sellner, 2021). Glucocorticoid drugs, such as methylprednisolone, are mainly used for the treatment of acute MS (Sato et al., 2012). Therefore, the need to develop new drugs and approaches for the treatment of MS persists. In recent years, with the in-depth study of MS, researchers have explored the mechanism of the immune system in inflammatory demyelination, neuronal injury, and myelin regeneration as well as the association between the immune system and CNS to find a better and more effective treatment strategy for MS. At present, research on MS therapeutic drugs is developing rapidly, and a number of these drugs are undergoing phase II and III clinical trials (Table 2) and basic study (Table 3).

**TABLE 2:** Drugs undergoing phase II and III clinical trials for the treatment of MS.

Product name	Clinical trials (Phase)	Pharmacological actions and Mechanisms	References
Rituximab	Ⅲ	CD20 monoclonal antibody; promotes the rapid extinction of B cells	Zhong et al. (2020); Chisari et al. (2021)
Laquinimod	Ⅲ	Regulates pro-inflammatory or anti-inflammatory cytokines secretion by Th1 and Th2 cells, and increase of brain-derived neurotrophic factor	Jolivel et al. (2013); Luhder et al. (2017)
Simvastatin	Ⅲ	Inhibits MHC II restricted antigen presentation, down-regulates T cell activation and proliferation, and induces the transition from proinflammatory Th1 to Th2	Chataway et al. (2014)
Ipilimumab	Ⅱ	Monoclonal antibody; effectively blocks the molecule of CTLA-4 and humanized antibody targeting cytokine LINGO-1	Gerdes et al. (2016)
Ibudilast	Ⅱ	Non-selective phosphodiesterase inhibitor; inhibits pro-inflammatory cytokines, promotes neurotrophic factors, and weakens activated glial cells	Fox et al. (2018); Naismith et al. (2021)
Mycophenolate mofetil	Ⅱ	Inhibits leukocyte apoptosis, weakens endothelial adhesion, and inhibits the migration of T and B cells	Michel et al. (2014); Xiao et al. (2014)
Amiloride	Ⅱ	Type-I acid-sensitive ion channel inhibitor; inhibits sodium and calcium influx into axonal and oligodendrocytes cells and protects neurons and myelin sheath from damage	Vergo et al. (2011)
Epigallocatechin-3-gallate	Ⅱ	Inhibits brain inflammation, neuronal injury, T cell proliferation, and TNF-α secretion in encephalitis	Spagnuolo et al. (2018)
Cannabinoids	Ⅱ	Cannabis receptor agonist; regulates the activation of cannabis receptors, resulting in a significant reduction of inflammatory cytokines and promoting the induction of anti-inflammatory cytokines	Al-Ghezi et al. (2019)
Erythropoietin	Ⅱ	Reduces the secretion of pro-inflammatory factors, maintains the integrity of the BBB, and increases the number of brain-derived neurotrophic factor positive cells and oligodendrocytes	Moransard et al. (2017); Gyetvai et al. (2018)
Flupirtine	Ⅱ	Activates inward rectifier potassium channels, plays a neuroprotective role and up-regulates Bcl-2 to increase neuronal survival	Shirani et al. (2016)
Lamotrigine	Ⅱ	Pressure-sensitive sodium channel antagonist; exerts neuroprotective effect by inhibiting intracellular calcium accumulation	Yang et al. (2015)
Riluzole	Ⅱ	Inhibits the release of glutamate at the ends of nerves and reduces axonal injury	Chataway et al. (2020)
Fluoxetine	Ⅱ	Inhibits the function of the Rho protein family, promotes myelin repair, and increases the level of anti-inflammatory factor IL-10 in serum	Milo (2015)
Oxcarbazepine	Ⅱ	A neuroprotective agent; inhibits microglial activity and neuronal sodium load	Cunniffe et al. (2021)

CD, cluster of differentiation; Th, helper T cells; MHC, major histocompatibility complex; CTLA-4, cytotoxic T cell antigen-4; LINGO-1, leucine-rich repeat and immunoglobulin domain-containing protein 1; TNF, tumor necrosis factor; BBB, blood–brain barrier; Rho, IL, interleukin.

**TABLE 3:** Drugs that are being studied for the treatment of MS in recent years.

Drugs name	Animal model	Pharmacological actions and Mechanisms	References
Rapamycin	EAE	Inhibits the activation of Th1 cells and superfunction of Th17 cells	Li et al. (2020)
Tacrolimus, FK506	EAE	Inhibits the dephosphorylation of nuclear factors in active T lymphocytes, reduces the secretion of IL-2, and inhibits the proliferation and activation of T cells	Kim et al. (2017)
Methylprednisolone	EAE	Regulates the gene expression of CD4^+^T lymphocytes, induces the proliferation of Treg cells, and reduces the secretion of proinflammatory cytokines	De Andres et al. (2018)
Metformin	CPZ	Activates AMPK pathway, induces oligodendrocyte and myelin proliferation, and reduces the proliferation of astrocytes and microglia	Largani et al. (2019)
Aspirin	EAE	Increases the level of IL-11, up-regulates Treg cells, and decreases Th1 and Th17 cells response	Pahan and Pahan (2019)
Trichostatin A	EAE	Histone deacetylase inhibitor; promotes the induction of peripheral T cell tolerance, reduces the migration of T cells to the spinal cord, and reduces neuronal injury	Jayaraman et al. (2017)
Ruxolitinib	EAE	Decreases the proportion of Th17 cells and the level of inflammatory factors, and increases the balance of Tregs and level of anti-inflammatory cytokines	Hosseini et al. (2021)
Tofacitinib	EAE	Inhibits DCs expression of costimulatory molecules, activates marker molecules and pro-inflammatory factors, inhibits antigen-specific T cell activation and differentiation, and reduces spinal cord leukocyte infiltration	Zhou et al. (2016)
Hydroxyfasudil	CPZ	Reduces microglial-mediated neuroinflammation and promotes the production of astrocyte-derived BDNF and the regeneration of NG2 oligodendrocyte precursor cells	Wang et al. (2019)
α-lipoic acid	EAE	Inhibits the proliferation of T lymphocytes and macrophages in the CNS and reduces axonal injury	Baldassari and Fox (2018)
Cornus iridoid glycoside	EAE	Limits the entry of T cells into the CNS and the activation of microglia, increases the expression of BDNF and mature oligodendrocytes, reduces OPC, inhibits brain JAK/STAT1/3, and reduces pro-inflammatory cytokines	Qu et al. (2019)
Icariin	CPZ	Improves the recovery of myelin, enhances the repair of NF200 positive axons, increases the number of mature oligodendrocytes in APC/Olig2, and prevents the loss of neuron-derived neurotrophic factors (such as NGF)	Zhang et al. (2017)
Icariin	EAE	Reduces microglia infiltration and spinal cord inflammation and demyelination, and inhibits Th1 and Th17 cells differentiation	Shen et al. (2015); Cong et al. (2020)
Pulsatilla saponin A3	EAE	Inhibits inflammatory Th1 and Th17 responses and transforms Th1 into Th2	Ip et al. (2015); Ip et al. (2017)
Dendrosomal nano-curcumin (DNC)	CPZ	Inhibits the activation of astrocytes and microglia and protects oligodendrocytes and myelin cells	Motavaf et al. (2020)
Bilobalide	EAE	Inhibits the infiltration of T cells and macrophages, reduces the expansion of neuroinflammation, and causes the apoptosis of oligodendrocytes in the CNS	Miao et al. (2020)
6-Gingerol	EAE	Inhibits the entry of inflammatory cells from the periphery into the CNS, inhibits the activation of DCs, and induces tolerance	Han et al. (2019)
Artemisinin	EAE	Inhibits inflammatory Th1 and Th17 responses and transforms immune responsive Th1 into Th2 cells	Khakzad et al. (2017)
9,10-Anhydrodehydroartemisinin	EAE	Reduces the levels of CNS and peripheral immune system infiltrating inflammatory cells Th1 and Th17	Lv et al. (2021)
Artesunate	EAE	Inhibits the migration of pathogenic T cells into the CNS	Thome et al. (2016)

EAE, experimental autoimmune encephalomyelitis; CPZ, cuprizone; Th, helper T cells; IL, interleukin; CD, cluster of differentiation; AMPK, adenosine monophosphate-activated protein kinase; DCs, dendritic cells; BDNF, brain-derived neurotrophic factor; CNS, central nervous system; OPC, oligodendrocyte progenitor cell; NF200, neurofilament 200 kDa; APC, antigen-presenting cells; Olig2, oligodendrocyte transcription factor-2; NGF, nerve growth factor.

### MS Drugs Acting on dendritic cells (DCs)

DCs are full-time antigen-presenting cells that do not only efficiently absorb, process, and present antigens as well as mediate antigen-specific immune responses but also regulate immune induction and maintain immune homeostasis. Increasing evidence has shown that DCs play an important role in the pathogenesis of MS and it is the balance cells between Th1/Th2 and Th17/Treg. MS is an autoimmune disease mediated by Th cells. Therefore, the treatment of MS with DCs as targets has become a research hotspot. DCs are divided into immature DCs, mature DCs, and semi-mature DCs according to their state of maturity. The main function of immature DCs is antigen presentation, whereas the main function of mature DCs is to induce T cell activation. Based on their phenotypic function, they can be divided into two categories: conventional DCs and plasmacytoid DCs (Macri et al., 2016; Rosa et al., 2020).

Kim et al. (Kim et al., 2016) found that minocycline-treated DCs (Mino-DCs) could induce the differentiation of Foxp3^+^ T cells with low expression of MHC II and costimulatory molecules and high expression of PDL-1. Injection of MOG_35-55_-shocked Mino-DCs into experimental autoimmune encephalomyelitis (EAE) mice could improve the clinical symptoms of EAE. Krivenko et al. (Krivenko et al., 2020) evaluated the effect of fluoxetine on the production of IL-6 and IL-1 β by DCs in MS and showed that the compound could inhibit the production of these cytokines, indicating that fluoxetine could exert an anti-inflammatory effect on MS by regulating the production of pro-inflammatory cytokines by DCs. Further, Chen et al. (Chen et al., 2018) showed that DCs treated with atorvastatin maintained a stable semi-phenotype and low levels of costimulatory molecules and pro-inflammatory cytokines. The drug significantly reduced disease activity in EAE mice, regulated Th17/Treg balance, significantly reduced Th17 cells, and increased regulatory T cells, which is expected to become a new strategy for the treatment of MS in the future. Additionally, Wang et al. (Wang et al., 2016) showed that daphnetin could significantly inhibit the reaction of Th1 and Th17 cells as well as the activation, maturation, and antigen presentation of DCs. At the same time, NF-κB signal decreased significantly in DCs treated with daphnetin accompanied by the induction of heme oxygenase-1 (a negative regulator of inflammatory signal). Mondal et al. (Mondal et al., 2018) have also reported that aspirin reduces the development of EAE driven by myelin basic protein-specific T cells, increases the amount of Foxp3 and IL-4 in T cells, and inhibits the differentiation of T cells into helper T cells (Th17 and Th1 cells).

### MS Drugs Acting on Astrocytes and Microglia

The exact etiology and pathogenesis of MS remain unclear. Its possible pathological features include the activation of astrocytes and microglia. A variety of pro-inflammatory and anti-inflammatory cytokines and chemokines secreted by activated astrocytes and microglia can be used as direct or indirect immune mediators or inflammatory mediators in the pathogenesis of MS (Yi et al., 2019). In addition, Magnus et al. (Magnus et al., 2004) suggested that both microglia and astrocytes could absorb apoptotic cells, and the phagocytosis of microglia and astrocytes is determined by the nearby microenvironment, both of which play important roles in the occurrence and development of MS.

EAE and toxic demyelination induced by cuprizone (CPZ) are commonly used in animal models of MS that are used to study demyelination and remyelination during the infiltration of inflammatory cells in the CNS. Wang et al. (Wang et al., 2020) showed that fasudil could inhibit microglial-mediated neuroinflammation and promote astrocyte-derived nerve growth factor and ciliary neurotrophic factor in CPZ-induced demyelination. Arsenic trioxide is used to treat a variety of autoimmune diseases. An et al. (An et al., 2020) showed that this compound could reduce demyelination, inflammation, microglial activation, and the expression of IL-2, IFN-γ, IL-1 β, IL-6, and TNF-α in EAE mice. It is expected to become a new drug for the treatment of MS. Honokiol, a nano-liposome developed by Hsiao et al. (Hsiao et al., 2020), is a drug that could reduce the number of IL-6^+^, Iba-1^+^ TNF^+^, Iba-1^+^ IL-12 p40^+^, and CD3^+^ IFN-γ+cells infiltrating the spinal cord and clearing the inhibitory effect of nanosome-encapsulated honokiol on the infiltration of activated microglia and Th1 cells into the spinal cord. Studies on microglia have shown that resveratrol-treated microglia can significantly inhibit the production of nitric oxide and TNF-α (Pallares et al., 2012). The same study emphasized the inhibitory effect of resveratrol on NF-κB in microglia (Nishikawa et al., 2015). In our laboratory, we found that cornel iridoid glycoside, icariin, and epimedium flavonoids could improve the symptoms of neurological damage in EAE and CPZ mice and inhibit the over-activation of microglia and astrocytes in the brain; thus, they may be potential effective drugs for the treatment of MS (Yin et al., 2012; Yin et al., 2014; Liang et al., 2015; Qu et al., 2016; Zhang et al., 2017; Qu et al., 2019).

### MS Drugs Acting on Oligodendrocytes

Oligodendrocytes wrap nerve fibers in the CNS with a special cell membrane to form a myelin sheath. In MS, the loss of myelin and oligodendrocytes impairs jumping signal transduction, leading to neuronal loss and dysfunction (Yeung et al., 2019). The limited ability of oligodendrocyte progenitor cells to differentiate into mature cells is the main reason for the low efficiency of myelin repair in the CNS. Oligodendrocytes are important cells for the regeneration of myelin sheath and axons. To ensure myelin regeneration, oligodendrocyte progenitor cells must migrate from the demyelinating area of the subependymal zone of the lateral ventricle and then mature into oligodendrocytes to promote myelin regeneration.

Manousi et al. (Manousi et al., 2021) identified some new small molecules that could promote oligodendrocyte differentiation, even in the presence of the oligodendrocyte differentiation inhibitor p57Kip2 and found subsets that could promote human oligodendrocyte genesis and myelin formation *in vitro*. Among them, danazol and parbendazole promote oligodendrocyte differentiation and myelin repair. It has been reported that astaxanthin has a protective effect against neurodegenerative diseases and can reduce CNS damage caused by oxidative stress. Lotfi et al. (Lotfi et al., 2021) showed that astaxanthin plays a beneficial role in reducing demyelination and oligodendrocyte death in an MS rat model. Astragalus polysaccharides are the main bioactive components of the astragalus membrane, which can prevent demyelination in EAE and CPZ mice. Ye et al. (Ye et al., 2021) showed that astragalus polysaccharides inhibited the dryness of neural stem cells and promoted the differentiation of neural stem cells into oligodendrocytes and neurons. Ghaiad et al. (Ghaiad et al., 2017) also showed that resveratrol could increase the expression of oligodendrocyte transcription factor-1 and promote myelin regeneration, which has a potential value in the treatment of MS.

In summary, the adaptive immune response T and B cells as well as myeloid cells of the innate immune system (dendritic cells, astrocytes, and microglia) are not only components of the CNS but also participate in the regulation of neuroimmune inflammatory response as immune helper cells in the CNS. Their roles in the occurrence and development of MS cannot be ignored. However, to date, the mechanism of their action in MS is not yet completely clear, and the therapeutic target is still vague. Therefore, the target of effective intervention in their pathogenic process, that is promoting neuroprotection, could be a feasible treatment to alleviate MS, which may also be the target of drug development in the future. However, although a few studies based on the above exist, further experimental and clinical evidence is needed, which is expected to provide new ideas and strategies for the prevention and treatment of MS.

### Summary

MS is characterized by peripheral and central inflammation, demyelination, and neurodegeneration. Although currently available MS treatments reduce the recurrence of the disease, they do not promote tolerance to myelin-specific T lymphocytes to ensure long-term protection against MS. Therefore, the treatment of MS is one of the biggest treatment challenges. To develop new and effective treatments for patients with MS, the mechanism of the disease needs to be fully clarified and understood, which may be multifactorial. However, the incomplete understanding of the pathogenesis of MS and lack of suitable animal models make it difficult to identify potential target pathways and new therapeutic drugs. Although the clinical use of some MS treatment drugs can alleviate the process of the disease, but they cannot completely cure MS and cause adverse reactions; thus, it is particularly important to actively seek safe and effective drugs for MS treatment. In summary, in view of the significant effects of drugs on autoimmunity and the regulation of the immune system, immunomodulatory drugs are expected to become candidates for the treatment of major autoimmune diseases. Although it is still a major challenge to develop effective strategies for the treatment of autoimmune diseases, such as MS, the complex immune regulation of drugs targeting various pathways in the treatment of autoimmune diseases is of great significance. Therefore, we have good reasons and are motivated to expand our exploration of drugs regulating the immune system to study and develop effective drugs for the treatment of MS.

## References

[B1] Aharoni R. (2013). The Mechanism of Action of Glatiramer Acetate in Multiple Sclerosis and Beyond. Autoimmun. Rev. 12 (5), 543–553. 10.1016/j.autrev.2012.09.005 23051633

[B2] Al-Ghezi Z. Z. Busbee P. B. Alghetaa H. Nagarkatti P. S. Nagarkatti M. (2019). Combination of Cannabinoids, Delta-9-Tetrahydrocannabinol (Thc) and Cannabidiol (Cbd), Mitigates Experimental Autoimmune Encephalomyelitis (Eae) by Altering the Gut Microbiome. Brain Behav. Immun. 82, 25–35. 10.1016/j.bbi.2019.07.028 31356922 PMC6866665

[B3] An K. Xue M.-J. Zhong J.-Y. Yu S.-N. Lan T.-S. Qi Z.-Q. (2020). Arsenic Trioxide Ameliorates Experimental Autoimmune Encephalomyelitis in C57BL/6 Mice by Inducing CD4+ T Cell Apoptosis. J. Neuroinflammation 17 (1), 147. 10.1186/s12974-020-01829-x 32375831 PMC7201567

[B4] Antonio García Merino J. (2014). Tratamiento actual de la esclerosis múltiple. Medicina Clínica 143 (Suppl. 3), 19–22. 10.1016/S0025-7753(15)30005-1 25732945

[B5] Bagherpour B. Salehi M. Jafari R. Bagheri A. Kiani-Esfahani A. Edalati M. (2018). Promising Effect of Rapamycin on Multiple Sclerosis. Mult. Scler. Relat. Disord. 26, 40–45. 10.1016/j.msard.2018.08.009 30219744

[B6] Baldassari L. E. Fox R. J. (2018). Therapeutic Advances and Challenges in the Treatment of Progressive Multiple Sclerosis. Drugs 78 (15), 1549–1566. 10.1007/s40265-018-0984-5 30255442

[B7] Bar-Or A. Pachner A. Menguy-Vacheron F. Kaplan J. Wiendl H. (2014). Teriflunomide and its Mechanism of Action in Multiple Sclerosis. Drugs 74 (6), 659–674. 10.1007/s40265-014-0212-x 24740824 PMC4003395

[B8] Beer K. Müller M. Hew-Winzeler A. M. Bont A. Maire P. You X. (2011). The Prevalence of Injection-Site Reactions with Disease-Modifying Therapies and Their Effect on Adherence in Patients with Multiple Sclerosis: An Observational Study. BMC Neurol. 11, 144. 10.1186/1471-2377-11-144 22074056 PMC3227610

[B9] Bigaut K. De Seze J. Collongues N. (2019). Ocrelizumab for the Treatment of Multiple Sclerosis. Expert Rev. Neurotherapeutics 19 (2), 97–108. 10.1080/14737175.2019.1561284 30570368

[B10] Bittner S. Afzali A. M. Wiendl H. Meuth S. G. (2014). Myelin Oligodendrocyte Glycoprotein (MOG35-55) Induced Experimental Autoimmune Encephalomyelitis (EAE) in C57bl/6 Mice. J. Vis. Exp. (86), 51275. 10.3791/51275 PMC4172026 24797125

[B11] Boster A. Nicholas J. Wu N. Yeh W.-S. Fay M. Edwards M. (2017). Comparative Effectiveness Research of Disease-Modifying Therapies for the Management of Multiple Sclerosis: Analysis of a Large Health Insurance Claims Database. Neurol. Ther. 6 (1), 91–102. 10.1007/s40120-017-0064-x 28211024 PMC5447557

[B12] Burns S. A. Lee Archer R. Chavis J. A. Tull C. A. Hensley L. L. Drew P. D. (2012). Mitoxantrone Repression of Astrocyte Activation: Relevance to Multiple Sclerosis. Brain Res. 1473, 236–241. 10.1016/j.brainres.2012.07.054 22884503 PMC3460523

[B13] Buron M. D. Kalincik T. Sellebjerg F. Sørensen P. S. Magyari M. (2021). Effect of Lateral Therapy Switches to Oral Moderate-Efficacy Drugs in Multiple Sclerosis: A Nationwide Cohort Study. J. Neurol. Neurosurg. Psychiatry 92 (5), 556–562. 10.1136/jnnp-2020-324869 33436501

[B14] Chataway J. De Angelis F. Connick P. Parker R. A. Plantone D. Doshi A. (2020). Efficacy of Three Neuroprotective Drugs in Secondary Progressive Multiple Sclerosis (Ms-Smart): A Phase 2b, Multiarm, Double-Blind, Randomised Placebo-Controlled Trial. Lancet Neurol. 19 (3), 214–225. 10.1016/S1474-4422(19)30485-5 31981516 PMC7029307

[B15] Chataway J. Schuerer N. Alsanousi A. Chan D. MacManus D. Hunter K. (2014). Effect of High-Dose Simvastatin on Brain Atrophy and Disability in Secondary Progressive Multiple Sclerosis (Ms-Stat): A Randomised, Placebo-Controlled, Phase 2 Trial. The Lancet 383 (9936), 2213–2221. 10.1016/S0140-6736(13)62242-4 24655729

[B16] Chen Z. Yang D. Peng X. Lin J. Su Z. Li J. (2018). Beneficial Effect of Atorvastatin-Modified Dendritic Cells Pulsed with Myelin Oligodendrocyte Glycoprotein Autoantigen on Experimental Autoimmune Encephalomyelitis. Neuroreport 29 (4), 317–327. 10.1097/WNR.0000000000000962 29394220

[B17] Chisari C. G. Sgarlata E. Arena S. Toscano S. Luca M. Patti F. (2021). Rituximab for the Treatment of Multiple Sclerosis: A Review. J. Neurol. 1–25. 10.1007/s00415-020-10362-z 33416999 PMC7790722

[B18] Cohan S. (2016). Therapeutic Efficacy of Monthly Subcutaneous Injection of Daclizumab in Relapsing Multiple Sclerosis. Btt 10, 119–138. 10.2147/BTT.S89218 PMC5026217 27672308

[B19] Cohen J. A. Barkhof F. Comi G. Hartung H.-P. Khatri B. O. Montalban X. (2010). Oral Fingolimod or Intramuscular Interferon for Relapsing Multiple Sclerosis. N. Engl. J. Med. 362 (5), 402–415. 10.1056/NEJMoa0907839 20089954

[B20] Cohen J. A. Khatri B. Barkhof F. Comi G. Hartung H.-P. Montalban X. (2016). Long-Term (Up to 4.5 Years) Treatment with Fingolimod in Multiple Sclerosis: Results from the Extension of the Randomised Transforms Study. J. Neurol. Neurosurg. Psychiatry 87 (5), 468–475. 10.1136/jnnp-2015-310597 26111826 PMC4853559

[B21] Comi G. Cohen J. A. Arnold D. L. Wynn D. Filippi M. Group F. S. (2011). Phase Iii Dose-Comparison Study of Glatiramer Acetate for Multiple Sclerosis. Ann. Neurol. 69 (1), 75–82. 10.1002/ana.22316 21280077

[B22] Confavreux C. Li D. K. Freedman M. S. Truffinet P. Benzerdjeb H. Wang D. (2012). Long-Term Follow-Up of a Phase 2 Study of Oral Teriflunomide in Relapsing Multiple Sclerosis: Safety and Efficacy Results up to 8.5 Years. Mult. Scler. 18 (9), 1278–1289. 10.1177/1352458512436594 22307384 PMC3573681

[B23] Confavreux C. O’Connor P. Comi G. Freedman M. S. Miller A. E. Olsson T. P. (2014). Oral Teriflunomide for Patients with Relapsing Multiple Sclerosis (Tower): A Randomised, Double-Blind, Placebo-Controlled, Phase 3 Trial. Lancet Neurol. 13 (3), 247–256. 10.1016/S1474-4422(13)70308-9 24461574

[B24] Cong H. Zhang M. Chang H. Du L. Zhang X. Yin L. (2020). Icariin Ameliorates the Progression of Experimental Autoimmune Encephalomyelitis by Down-Regulating the Major Inflammatory Signal Pathways in a Mouse Relapse-Remission Model of Multiple Sclerosis. Eur. J. Pharmacol. 885, 173523. 10.1016/j.ejphar.2020.173523 32871176

[B25] Correale J. Gaitán M. I. Ysrraelit M. C. Fiol M. P. (2017). Progressive Multiple Sclerosis: From Pathogenic Mechanisms to Treatment. Brain 140 (3), 527–546. 10.1093/brain/aww258 27794524

[B26] Cunniffe N. Vuong K. A. Ainslie D. Baker D. Beveridge J. Bickley S. (2021). Systematic Approach to Selecting Licensed Drugs for Repurposing in the Treatment of Progressive Multiple Sclerosis. J. Neurol. Neurosurg. Psychiatry 92 (3), 295–302. 10.1136/jnnp-2020-324286 33184094

[B27] De Andres C. García M. I. Goicoechea H. Martínez-Ginés M. L. García-Domínguez J. M. Martín M. L. (2018). Genes Differentially Expressed by Methylprednisolone In Vivo in Cd4 T Lymphocytes from Multiple Sclerosis Patients: Potential Biomarkers. Pharmacogenomics J. 18 (1), 98–105. 10.1038/tpj.2016.71 27670768

[B28] De Angelis F. Plantone D. Chataway J. (2018). Pharmacotherapy in Secondary Progressive Multiple Sclerosis: An Overview. CNS Drugs 32 (6), 499–526. 10.1007/s40263-018-0538-0 29968175

[B29] Dendrou C. A. Fugger L. Friese M. A. (2015). Immunopathology of Multiple Sclerosis. Nat. Rev. Immunol. 15 (9), 545–558. 10.1038/nri3871 26250739

[B30] Dobson R. Giovannoni G. (2019). Multiple Sclerosis - A Review. Eur. J. Neurol. 26 (1), 27–40. 10.1111/ene.13819 30300457

[B31] Edan G. Morrissey S. Le Page E. (2004). Rationale for the Use of Mitoxantrone in Multiple Sclerosis. J. Neurol. Sci. 223 (1), 35–39. 10.1016/j.jns.2004.04.017 15261558

[B33] Filippini G. Del Giovane C. Clerico M. Beiki O. Mattoscio M. Piazza F. (2017). Treatment with Disease-Modifying Drugs for People with a First Clinical Attack Suggestive of Multiple Sclerosis. Cochrane Database Syst. Rev. 4 (4), CD012200. 10.1002/14651858.CD012200.pub2 28440858 PMC6478290

[B34] Findling O. Hauer L. Pezawas T. Rommer P. S. Struhal W. Sellner J. (2020). Cardiac Autonomic Dysfunction in Multiple Sclerosis: A Systematic Review of Current Knowledge and Impact of Immunotherapies. Jcm 9 (2), 335. 10.3390/jcm9020335 PMC7073977 31991711

[B35] Findling O. Sellner J. (2021). Second-Generation Immunotherapeutics in Multiple Sclerosis: Can We Discard Their Precursors?. Drug Discov. Today 26 (2), 416–428. 10.1016/j.drudis.2020.11.022 33248250

[B36] Fox R. J. Coffey C. S. Conwit R. Cudkowicz M. E. Gleason T. Goodman A. (2018). Phase 2 Trial of Ibudilast in Progressive Multiple Sclerosis. N. Engl. J. Med. 379 (9), 846–855. 10.1056/NEJMoa1803583 30157388 PMC6172944

[B37] Gajofatto A. Benedetti M. D. (2015). Treatment Strategies for Multiple Sclerosis: When to Start, When to Change, When to Stop? Wjcc 3 (7), 545–555. 10.12998/wjcc.v3.i7.545 26244148 PMC4517331

[B38] Gerdes L. A. Held K. Beltrán E. Berking C. Prinz J. C. Junker A. (2016). Ctla4 as Immunological Checkpoint in the Development of Multiple Sclerosis. Ann. Neurol. 80 (2), 294–300. 10.1002/ana.24715 27351142 PMC5129566

[B39] Ghaiad H. R. Nooh M. M. El-Sawalhi M. M. Shaheen A. A. (2017). Resveratrol Promotes Remyelination in Cuprizone Model of Multiple Sclerosis: Biochemical and Histological Study. Mol. Neurobiol. 54 (5), 3219–3229. 10.1007/s12035-016-9891-5 27067589

[B40] Gold R. Stefoski D. Selmaj K. Havrdova E. Hurst C. Holman J. (2016). Pregnancy Experience: Nonclinical Studies and Pregnancy Outcomes in the Daclizumab Clinical Study Program. Neurol. Ther. 5 (2), 169–182. 10.1007/s40120-016-0048-2 27411694 PMC5130915

[B41] Gold R. Wolinsky J. S. (2011). Pathophysiology of Multiple Sclerosis and the Place of Teriflunomide. Acta Neurol. Scand. 124 (2), 75–84. 10.1111/j.1600-0404.2010.01444.x 20880295

[B42] Gross C. C. Ahmetspahic D. Ruck T. Schulte-Mecklenbeck A. Schwarte K. Jörgens S. (2016). Alemtuzumab Treatment Alters Circulating Innate Immune Cells in Multiple Sclerosis. Neurol. Neuroimmunol Neuroinflamm 3 (6), e289. 10.1212/NXI.0000000000000289 27766281 PMC5063395

[B43] Gyetvai G. Roe C. Heikal L. Ghezzi P. Mengozzi M. (2018). Leukemia Inhibitory Factor Inhibits Erythropoietin-Induced Myelin Gene Expression in Oligodendrocytes. Mol. Med. 24 (1), 51. 10.1186/s10020-018-0052-3 30261841 PMC6161334

[B44] Han J. J. Li X. Ye Z. Q. Lu X. Y. Yang T. Tian J. (2019). Treatment with 6‐Gingerol Regulates Dendritic Cell Activity and Ameliorates the Severity of Experimental Autoimmune Encephalomyelitis. Mol. Nutr. Food Res. 63 (18), e1801356. 10.1002/mnfr.201801356 31313461

[B45] Hosseini A. Gharibi T. Mohammadzadeh A. Ebrahimi-Kalan A. Jadidi-Niaragh F. Babaloo Z. (2021). Ruxolitinib Attenuates Experimental Autoimmune Encephalomyelitis (Eae) Development as Animal Models of Multiple Sclerosis (Ms). Life Sci. 276, 119395. 10.1016/j.lfs.2021.119395 33781828

[B46] Hsiao Y.-P. Chen H.-T. Liang Y.-C. Wang T.-E. Huang K.-H. Hsu C.-C. (2020). Development of Nanosome-Encapsulated Honokiol for Intravenous Therapy against Experimental Autoimmune Encephalomyelitis. Ijn 15, 17–29. 10.2147/IJN.S214349 32021162 PMC6954093

[B47] Hupperts R. Smolders J. Vieth R. Holmøy T. Marhardt K. Schluep M. (2019). Randomized Trial of Daily High-Dose Vitamin D3 in Patients with RRMS Receiving Subcutaneous Interferon β-1a. Neurology 93 (20), e1906–e1916. 10.1212/WNL.0000000000008445 31594857 PMC6946471

[B48] Imeri F. Stepanovska Tanturovska B. Zivkovic A. Enzmann G. Schwalm S. Pfeilschifter J. (2021). Novel Compounds with Dual S1p Receptor Agonist and Histamine H3 Receptor Antagonist Activities Act Protective in a Mouse Model of Multiple Sclerosis. Neuropharmacology 186, 108464. 10.1016/j.neuropharm.2021.108464 33460688

[B49] Ip F. C. F. Ng Y. P. Or T. C. T. Sun P. Fu G. Li J. Y. H. (2017). Anemoside A3 Ameliorates Experimental Autoimmune Encephalomyelitis by Modulating T Helper 17 Cell Response. PLoS One 12 (7), e0182069. 10.1371/journal.pone.0182069 28759648 PMC5536310

[B50] Ip F. C. Fu W.-Y. Cheng E. Y. Tong E. P. Lok K.-C. Liang Y. (2015). Anemoside A3 Enhances Cognition Through the Regulation of Synaptic Function and Neuroprotection. Neuropsychopharmacol 40 (8), 1877–1887. 10.1038/npp.2015.37 PMC4839511 25649278

[B51] Jain A. Jain S. (2008). Pegylation: An Approach for Drug Delivery. A Review. Crit. Rev. Ther. Drug Carrier Syst. 25 (5), 403–447. 10.1615/critrevtherdrugcarriersyst.v25.i5.10 19062633

[B52] Jayaraman A. Soni A. Prabhakar B. S. Holterman M. Jayaraman S. (2017). The Epigenetic Drug Trichostatin a Ameliorates Experimental Autoimmune Encephalomyelitis via T Cell Tolerance Induction and Impaired Influx of T Cells into the Spinal Cord. Neurobiol. Dis. 108, 1–12. 10.1016/j.nbd.2017.07.015 28736194

[B53] Jeffery D. R. Herndon R. (2004). Review of Mitoxantrone in the Treatment of Multiple Sclerosis. Neurology 63 (12 Suppl. 6), S19–S24. 10.1212/wnl.63.12_suppl_6.s19 15623665

[B54] Jolivel V. Luessi F. Masri J. Kraus S. H. P. Hubo M. Poisa-Beiro L. (2013). Modulation of Dendritic Cell Properties by Laquinimod as a Mechanism for Modulating Multiple Sclerosis. Brain 136 (Pt 4), 1048–1066. 10.1093/brain/awt023 23518712

[B55] Khakzad M. R. Ganji A. Ariabod V. Farahani I. (2017). Artemisinin Therapeutic Efficacy in the Experimental Model of Multiple Sclerosis. Immunopharmacology and Immunotoxicology 39 (6), 348–353. 10.1080/08923973.2017.1379087 28952817

[B56] Kidd T. Carey N. Mold F. Westwood S. Miklaucich M. Konstantara E. (2017). A Systematic Review of the Effectiveness of Self-Management Interventions in People with Multiple Sclerosis at Improving Depression, Anxiety and Quality of Life. PLoS One 12 (10), e0185931. 10.1371/journal.pone.0185931 29020113 PMC5636105

[B57] Kim M.-J. Sung J.-J. Kim S. H. Kim J.-M. Jeon G. S. Mun S.-K. (2017). The Anti-inflammatory Effects of Oral-Formulated Tacrolimus in Mice with Experimental Autoimmune Encephalomyelitis. J. Korean Med. Sci. 32 (9), 1502–1507. 10.3346/jkms.2017.32.9.1502 28776347 PMC5546971

[B58] Kim N. Park C.-S. Im S.-A. Kim J.-W. Lee J.-H. Park Y.-J. (2016). Minocycline Promotes the Generation of Dendritic Cells with Regulatory Properties. Oncotarget 7 (33), 52818–52831. 10.18632/oncotarget.10810 27463004 PMC5288151

[B59] Klotz L. Havla J. Schwab N. Hohlfeld R. Barnett M. Reddel S. (2019). Risks and Risk Management in Modern Multiple Sclerosis Immunotherapeutic Treatment. Ther. Adv. Neurol. Disord. 12, 175628641983657. 10.1177/1756286419836571 PMC6444778 30967901

[B60] Krivenko L. V. Sviridova A. A. Melnikov M. V. Rogovskii V. S. Boyko A. N. Pashenkov M. V. (2020). The Influence of Fluoxetine on Interleukin-6 and Interleukin-1β Production by Dendritic Cells in Multiple Sclerosis In Vitro. Z. Nevrol. Psikhiatr. Im. S.S. Korsakova 120 (7), 67–72. 10.17116/jnevro202012007267 32844633

[B61] Krysko K. M. Graves J. S. Rensel M. Weinstock‐Guttman B. Rutatangwa A. Aaen G. (2020). Real‐World Effectiveness of Initial Disease‐Modifying Therapies in Pediatric Multiple Sclerosis. Ann. Neurol. 88 (1), 42–55. 10.1002/ana.25737 32267005

[B62] La Mantia L. Di Pietrantonj C. Rovaris M. Rigon G. Frau S. Berardo F. (2015). Comparative Efficacy of Interferon β Versus Glatiramer Acetate for Relapsing-Remitting Multiple Sclerosis. J. Neurol. Neurosurg. Psychiatry 86 (9), 1016–1020. 10.1136/jnnp-2014-309243 25550414

[B63] La Mantia L. Milanese C. Mascoli N. D’Amico R. Weinstock-Guttman B. (2007). Cyclophosphamide for Multiple Sclerosis. Cochrane Database Syst. Rev. 2007 (1), CD002819. 10.1002/14651858.CD002819.pub2 PMC8078225 17253481

[B64] Lamb Y. N. (2020). Ozanimod: First Approval. Drugs 80 (8), 841–848. 10.1007/s40265-020-01319-7 32385738

[B65] Largani S. H. H. Borhani-Haghighi M. Pasbakhsh P. Mahabadi V. P. Nekoonam S. Shiri E. (2019). Oligoprotective Effect of Metformin Through the Ampk-dependent on Restoration of Mitochondrial Hemostasis in the Cuprizone-Induced Multiple Sclerosis Model. J. Mol. Hist. 50 (3), 263–271. 10.1007/s10735-019-09824-0 31016544

[B66] Li X.-l. Zhang B. Liu W. Sun M.-j. Zhang Y.-l. Liu H. (2020). Rapamycin Alleviates the Symptoms of Multiple Sclerosis in Experimental Autoimmune Encephalomyelitis (Eae) Through Mediating the Tam-Tlrs-Socs Pathway. Front. Neurol. 11, 590884. 10.3389/fneur.2020.590884 33329339 PMC7728797

[B67] Liang M. Chen Y. Zhang L. Li L. Chen G. Yin L. (2015). Epimedium Flavonoids Ameliorate Neuropathological Changes and Increases Igf-1 Expression in C57bl/6 Mice Exposed to Cuprizone. Neurochem. Res. 40 (3), 492–500. 10.1007/s11064-014-1490-0 25663299

[B68] Lombardo S. D. Mazzon E. Basile M. S. Campo G. Corsico F. Presti M. (2019). Modulation of Tetraspanin 32 (Tspan32) Expression in T Cell-Mediated Immune Responses and in Multiple Sclerosis. Ijms 20 (18), 4323. 10.3390/ijms20184323 PMC6770290 31487788

[B69] Lotfi A. Soleimani M. Ghasemi N. (2021). Astaxanthin Reduces Demyelination and Oligodendrocytes Death in a Rat Model of Multiple Sclerosis. Cell J 22 (4), 565–571. 10.22074/cellj.2021.6999 32347051 PMC7211289

[B70] Lühder F. Kebir H. Odoardi F. Litke T. Sonneck M. Alvarez J. I. (2017). Laquinimod Enhances Central Nervous System Barrier Functions. Neurobiol. Dis. 102, 60–69. 10.1016/j.nbd.2017.02.002 28235673

[B71] Lv J. Zhuang W. Zhang Y. Xie L. Xiang Z. Zhao Q. (2021). 9,10-Anhydrodehydroartemisinin Attenuates Experimental Autoimmune Encephalomyelitis by Inhibiting Th1 and Th17 Cell Differentiation. Inflammation. 10.1007/s10753-021-01456-5 33788130

[B72] Macri C. Dumont C. Johnston A. P. Mintern J. D. (2016). Targeting Dendritic Cells: A Promising Strategy to Improve Vaccine Effectiveness. Clin. Trans. Immunol. 5 (3), e66. 10.1038/cti.2016.6 PMC4815026 27217957

[B73] Magnus T. Korn T. Jung S. (2004). Chronically Stimulated Microglial Cells Do No Longer Alter Their Immune Functions in Response to the Phagocytosis of Apoptotic Cells. J. Neuroimmunology 155 (1-2), 64–72. 10.1016/j.jneuroim.2004.06.002 15342197

[B74] Manousi A. Göttle P. Reiche L. Cui Q.-L. Healy L. M. Akkermann R. (2021). Identification of Novel Myelin Repair Drugs by Modulation of Oligodendroglial Differentiation Competence. EBioMedicine 65, 103276. 10.1016/j.ebiom.2021.103276 33714029 PMC7970057

[B75] Martinelli V. Radaelli M. Straffi L. Rodegher M. Comi G. (2009). Mitoxantrone: Benefits and Risks in Multiple Sclerosis Patients. Neurol. Sci. 30 (Suppl. 2), 167–170. 10.1007/s10072-009-0142-7 19882368

[B76] Matko S. Akgün K. Tonn T. Ziemssen T. Odendahl M. (2020). Antigen-Shift in Varicella-Zoster Virus-specific T-Cell Immunity over the Course of Fingolimod-Treatment in Relapse-Remitting Multiple Sclerosis Patients. Mult. Scler. Relat. Disord. 38, 101859. 10.1016/j.msard.2019.101859 31855843

[B77] Menge T. Rehberg-Weber K. Taipale K. Nastos I. Jauss M. (2021). Peginterferon Beta-1a Was Associated with High Adherence and Satisfaction in Patients with Multiple Sclerosis in a German Real-World Study. Ther. Adv. Neurol. Disord. 14, 175628642110004. 10.1177/17562864211000461 PMC7983429 33796146

[B78] Menzin J. Caon C. Nichols C. White L. A. Friedman M. Pill M. W. (2013). Narrative Review of the Literature on Adherence to Disease-Modifying Therapies Among Patients with Multiple Sclerosis. Jmcp 19 (1 Suppl. A), S24–S40. 10.18553/jmcp.2013.19.s1.S24 23383731 PMC10437533

[B79] Miao Q. Zhang X.-X. Han Q.-X. Ren S.-S. Sui R.-X. Yu J.-W. (2020). The Therapeutic Potential of Bilobalide on Experimental Autoimmune Encephalomyelitis (Eae) Mice. Metab. Brain Dis. 35 (5), 793–807. 10.1007/s11011-020-00555-w 32215835

[B80] Michel L. Vukusic S. De Seze J. Ducray F. Ongagna J. C. Lefrere F. (2014). Mycophenolate Mofetil in Multiple Sclerosis: A Multicentre Retrospective Study on 344 Patients. J. Neurol. Neurosurg. Psychiatry 85 (3), 279–283. 10.1136/jnnp-2013-305298 23704316

[B81] Milo R. (2015). Effectiveness of Multiple Sclerosis Treatment with Current Immunomodulatory Drugs. Expert Opin. Pharmacother. 16 (5), 659–673. 10.1517/14656566.2015.1002769 25582634

[B82] Miravalle A. A. Katz J. Robertson D. Hayward B. Harlow D. E. Lebson L. A. (2021). Click-Ms and Master-2 Phase Iv Trial Design: Cladribine Tablets in Suboptimally Controlled Relapsing Multiple Sclerosis. Neurodegenerative Dis. Manag. 11 (2), 99–111. 10.2217/nmt-2020-0059 33517769

[B83] Mohr D. C. Boudewyn A. C. Likosky W. Levine E. Goodkin D. E. (2001). Injectable Medication for the Treatment of Multiple Sclerosis: The Influence of Self-Efficacy Expectations and Infection Anxiety on Adherence and Ability to Self-Inject. Ann. Behav. Med. 23 (2), 125–132. 10.1207/S15324796ABM2302_7 11394554

[B84] Mondal S. Jana M. Dasarathi S. Roy A. Pahan K. (2018). Aspirin Ameliorates Experimental Autoimmune Encephalomyelitis Through Interleukin-11-Mediated Protection of Regulatory T Cells. Sci. Signal. 11 (558), eaar8278. 10.1126/scisignal.aar8278 30482850 PMC6325078

[B85] Moransard M. Bednar M. Frei K. Gassmann M. Ogunshola O. O. (2017). Erythropoietin Reduces Experimental Autoimmune Encephalomyelitis Severity via Neuroprotective Mechanisms. J. Neuroinflammation 14 (1), 202. 10.1186/s12974-017-0976-5 29029628 PMC5640948

[B86] Motavaf M. Sadeghizadeh M. Babashah S. Zare L. Javan M. (2020). Protective Effects of a Nano-Formulation of Curcumin Against Cuprizone-Induced Demyelination in the Mouse Corpus Callosum. Iranian J. Pharm. Res. 19 (3), 310–320. 10.22037/ijpr.2020.112952.14033 PMC7758014 33680032

[B87] Myhr K.-M. (2008). Diagnosis and Treatment of Multiple Sclerosis. Acta Neurol. Scand. 117, 12–21. 10.1111/j.1600-0404.2008.01026.x 18439216

[B88] Naismith R. T. Bermel R. A. Coffey C. S. Goodman A. D. Fedler J. Kearney M. (2021). Effects of Ibudilast on Mri Measures in the Phase 2 Sprint-Ms Study. Neurology 96 (4), e491–e500. 10.1212/WNL.0000000000011314 33268562 PMC7905793

[B89] Naismith R. T. Wolinsky J. S. Wundes A. LaGanke C. Arnold D. L. Obradovic D. (2020a). Diroximel Fumarate (Drf) in Patients with Relapsing-Remitting Multiple Sclerosis: Interim Safety and Efficacy Results from the Phase 3 Evolve-Ms-1 Study. Mult. Scler. 26 (13), 1729–1739. 10.1177/1352458519881761 31680631 PMC7604551

[B90] Naismith R. T. Wundes A. Wundes A. Ziemssen T. Jasinska E. Freedman M. S. (2020b). Diroximel Fumarate Demonstrates an Improved Gastrointestinal Tolerability Profile Compared with Dimethyl Fumarate in Patients with Relapsing-Remitting Multiple Sclerosis: Results from the Randomized, Double-Blind, Phase Iii Evolve-Ms-2 Study. CNS Drugs 34 (2), 185–196. 10.1007/s40263-020-00700-0 31953790 PMC7018784

[B91] Nishikawa K. Iwaya K. Kinoshita M. Fujiwara Y. Akao M. Sonoda M. (2015). Resveratrol Increases CD68+Kupffer Cells Colocalized with Adipose Differentiation-Related Protein and Ameliorates High-Fat-Diet-Induced Fatty Liver in Mice. Mol. Nutr. Food Res. 59 (6), 1155–1170. 10.1002/mnfr.201400564 25677089

[B92] O'Connor P. Wolinsky J. S. Confavreux C. Comi G. Kappos L. Olsson T. P. (2011). Randomized Trial of Oral Teriflunomide for Relapsing Multiple Sclerosis. N. Engl. J. Med. 365 (14), 1293–1303. 10.1056/NEJMoa1014656 21991951

[B93] Oh J. Vidal-Jordana A. Montalban X. (2018). Multiple Sclerosis: Clinical Aspects. Curr. Opin. Neurol. 31 (6), 752–759. 10.1097/WCO.0000000000000622 30300239

[B94] Pahan S. Pahan K. (2019). Mode of Action of Aspirin in Experimental Autoimmune Encephalomyelitis. DNA Cel Biol. 38 (7), 593–596. 10.1089/dna.2019.4814 PMC6628982 31140860

[B95] Pallarès V. Calay D. Cedó L. Castell-Auví A. Raes M. Pinent M. (2012). Enhanced Anti-inflammatory Effect of Resveratrol and Epa in Treated Endotoxin-Activated Raw 264.7 Macrophages. Br. J. Nutr. 108 (9), 1562–1573. 10.1017/S0007114511007057 22221545

[B96] Patel A. Sul J. Gordon M. L. Steinklein J. Sanguinetti S. Pramanik B. (2021). Progressive Multifocal Leukoencephalopathy in a Patient with Progressive Multiple Sclerosis Treated with Ocrelizumab Monotherapy. JAMA Neurol. 78 (6), 736–740. 10.1001/jamaneurol.2021.0627 33724354 PMC7967248

[B97] Paterka M. Siffrin V. Voss J. O. Werr J. Hoppmann N. Gollan R. (2016). Gatekeeper Role of Brain Antigen‐presenting CD11c + Cells in Neuroinflammation. EMBO J. 35 (1), 89–101. 10.15252/embj.201591488 26612827 PMC4718005

[B98] Patti F. Lo Fermo S. (2011). Lights and Shadows of Cyclophosphamide in the Treatment of Multiple Sclerosis. Autoimmune Dis. 2011, 1–14. 10.4061/2011/961702 PMC3087413 21547093

[B99] Patti F. (2010). Optimizing the Benefit of Multiple Sclerosis Therapy: The Importance of Treatment Adherence. Ppa 4, 1–9. 10.2147/ppa.s8230 20165593 PMC2819898

[B100] Pavelek Z. Sobíšek L. Šarláková J. Potužník P. Peterka M. Štětkárová I. (2020). Comparison of Therapies in Ms Patients After the First Demyelinating Event in Real Clinical Practice in the Czech Republic: Data from the National Registry Remus. Front. Neurol. 11, 593527. 10.3389/fneur.2020.593527 33510704 PMC7835499

[B101] Qu Z. Zheng N. Wei Y. Chen Y. Zhang Y. Zhang M. (2019). Effect of Cornel Iridoid Glycoside on Microglia Activation Through Suppression of the Jak/Stat Signalling Pathway. J. Neuroimmunology 330, 96–107. 10.1016/j.jneuroim.2019.01.014 30852182

[B102] Qu Z. Zheng N. Zhang Y. Zhang L. Liu J. Wang Q. (2016). Preventing the Bdnf and Ngf Loss Involved in the Effects of Cornel Iridoid Glycoside on Attenuation of Experimental Autoimmune Encephalomyelitis in Mice. Neurol. Res. 38 (9), 831–837. 10.1080/01616412.2016.1200766 27373350

[B103] Rommer P. S. Zettl U. K. (2018). Managing the Side Effects of Multiple Sclerosis Therapy: Pharmacotherapy Options for Patients. Expert Opin. Pharmacother. 19 (5), 483–498. 10.1080/14656566.2018.1446944 29528247

[B104] Rosa F. Pires C. Zimmermannova O. Pereira C.-F. (2020). Direct Reprogramming of Mouse Embryonic Fibroblasts to Conventional Type 1 Dendritic Cells by Enforced Expression of Transcription Factors. Bio-protocol 10 (10), e3619. 10.21769/BioProtoc.3619 33659292 PMC7842401

[B105] Sato D. Callegaro D. Lana-Peixoto M. A. Fujihara K. (2012). Brazilian Committee for, T., and Research in Multiple, S. (Treatment of Neuromyelitis Optica: An Evidence Based Review. Arq. Neuro-psiquiatr. 70 (1), 59–66. 10.1590/s0004-282x2012000100012 22218475

[B106] Shahi S. K. Jensen S. N. Murra A. C. Tang N. Guo H. Gibson-Corley K. N. (2020). Human Commensal Prevotella Histicola Ameliorates Disease as Effectively as Interferon-Beta in the Experimental Autoimmune Encephalomyelitis. Front. Immunol. 11, 578648. 10.3389/fimmu.2020.578648 33362764 PMC7759500

[B107] Shen K. Reichelt M. Kyauk R. V. Ngu H. Shen Y.-A. A. Foreman O. (2021). Multiple Sclerosis Risk Gene Mertk Is Required for Microglial Activation and Subsequent Remyelination. Cel Rep. 34 (10), 108835. 10.1016/j.celrep.2021.108835 33691116

[B108] Shen R. Deng W. Li C. Zeng G. (2015). A Natural Flavonoid Glucoside Icariin Inhibits Th1 and Th17 Cell Differentiation and Ameliorates Experimental Autoimmune Encephalomyelitis. Int. Immunopharmacology 24 (2), 224–231. 10.1016/j.intimp.2014.12.015 25528476

[B109] Shirani A. Okuda D. T. Stüve O. (2016). Therapeutic Advances and Future Prospects in Progressive Forms of Multiple Sclerosis. Neurotherapeutics 13 (1), 58–69. 10.1007/s13311-015-0409-z 26729332 PMC4720678

[B110] Song J. Y. Griffin J. D. Larson N. R. Christopher M. A. Middaugh C. R. Berkland C. J. (2020). Synthetic Cationic Autoantigen Mimics Glatiramer Acetate Persistence at the Site of Injection and Is Efficacious Against Experimental Autoimmune Encephalomyelitis. Front. Immunol. 11, 603029. 10.3389/fimmu.2020.603029 33537031 PMC7848024

[B111] Spagnuolo C. Moccia S. Russo G. L. (2018). Anti-Inflammatory Effects of Flavonoids in Neurodegenerative Disorders. Eur. J. Med. Chem. 153, 105–115. 10.1016/j.ejmech.2017.09.001 28923363

[B112] Spampinato S. F. Merlo S. Sano Y. Kanda T. Sortino M. A. (2021). Protective Effect of the Sphingosine-1 Phosphate Receptor Agonist Siponimod on Disrupted Blood Brain Barrier Function. Biochem. Pharmacol. 186, 114465. 10.1016/j.bcp.2021.114465 33577891

[B113] Teymoori-Rad M. Sahraian M. A. Mokhtariazad T. Nejati A. Mozdabadi R. S. K. Amiri M. M. (2021). Illuminating the In Vitro Effects of Epstein-Barr Virus and Vitamin D on Immune Response in Multiple Sclerosis Patients. J. Neurovirol. 27 (2), 260–271. 10.1007/s13365-021-00951-7 33666884

[B114] Thomé R. de Carvalho A. C. Alves da Costa T. Ishikawa L. L. W. Fraga-Silva T. F. d. C. Sartori A. (2016). Artesunate Ameliorates Experimental Autoimmune Encephalomyelitis by Inhibiting Leukocyte Migration to the Central Nervous System. CNS Neurosci. Ther. 22 (8), 707–714. 10.1111/cns.12561 27165523 PMC6492826

[B32] U.S. Food and Drug Administration (2020). FDA·Zeposia® (Ozanimod) Capsules, for Oral Use [EB/OL]. Available at: https://www.accessdata.fda.gov/drugsatfda_docs/label/2020/209899s000lbl.pdf.

[B115] van Dijkman S. C. de Jager N. C. B. Rauwé W. M. Danhof M. Della Pasqua O. (2018). Effect of Age-Related Factors on the Pharmacokinetics of Lamotrigine and Potential Implications for Maintenance Dose Optimisation in Future Clinical Trials. Clin. Pharmacokinet. 57 (8), 1039–1053. 10.1007/s40262-017-0614-5 29363050

[B116] Vergo S. Craner M. J. Etzensperger R. Attfield K. Friese M. A. Newcombe J. (2011). Acid-Sensing Ion Channel 1 Is Involved in Both Axonal Injury and Demyelination in Multiple Sclerosis and its Animal Model. Brain 134 (Pt 2), 571–584. 10.1093/brain/awq337 21233144

[B117] Wang D. Lu Z. Zhang H. Jin S.-F. Yang H. Li Y.-M. (2016). Daphnetin Alleviates Experimental Autoimmune Encephalomyelitis via Regulating Dendritic Cell Activity. CNS Neurosci. Ther. 22 (7), 558–567. 10.1111/cns.12537 27013083 PMC6492777

[B118] Wang J. Sui R.-X. Miao Q. Wang Q. Song L.-J. Yu J.-Z. (2019). RETRACTED: Hydroxyfasudil Alleviates Demyelination Through the Inhibition of MOG Antibody and Microglia Activation in Cuprizone Mouse Model. Clin. Immunol. 201, 35–47. 10.1016/j.clim.2019.01.006 30660624

[B119] Wang J. Sui R. X. Miao Q. Wang Q. Song L. J. Yu J. Z. (2020). Retracted: Effect of Fasudil on Remyelination Following Cuprizone‐induced Demyelination. CNS Neurosci. Ther. 26 (1), 76–89. 10.1111/cns.13154 31124292 PMC6930827

[B120] Wittling M. C. Cahalan S. R. Levenson E. A. Rabin R. L. (2020). Shared and Unique Features of Human Interferon-Beta and Interferon-Alpha Subtypes. Front. Immunol. 11, 605673. 10.3389/fimmu.2020.605673 33542718 PMC7850986

[B121] Wolinsky J. S. Narayana P. A. Nelson F. Datta S. O’Connor P. Confavreux C. (2013). Magnetic Resonance Imaging Outcomes from a Phase Iii Trial of Teriflunomide. Mult. Scler. 19 (10), 1310–1319. 10.1177/1352458513475723 23447359

[B122] Xiao Y. Huang J. Luo H. Wang J. (2014). Mycophenolate Mofetil for Relapsing-Remitting Multiple Sclerosis. Cochrane Database Syst. Rev. (2), CD010242. 10.1002/14651858.CD010242.pub2 24505016 PMC10875409

[B123] Yamout B. I. Alroughani R. (2018). Multiple Sclerosis. Semin. Neurol. 38 (2), 212–225. 10.1055/s-0038-1649502 29791948

[B124] Yang C. Hao Z. Zhang L. Zeng L. Wen J. (2015). Sodium Channel Blockers for Neuroprotection in Multiple Sclerosis. Cochrane Database Syst. Rev. 10, CD010422. 10.1002/14651858.CD010422.pub2 PMC9242538 26486929

[B125] Ye N. Cruz J. Peng X. Ma J. Zhang A. Cheng X. (2021). Remyelination Is Enhanced by Astragalus Polysaccharides Through Inducing the Differentiation of Oligodendrocytes from Neural Stem Cells in Cuprizone Model of Demyelination. Brain Res. 1763, 147459. 10.1016/j.brainres.2021.147459 33794147

[B126] Yeung M. S. Y. Djelloul M. Steiner E. Bernard S. Salehpour M. Possnert G. (2019). Dynamics of Oligodendrocyte Generation in Multiple Sclerosis. Nature 566 (7745), 538–542. 10.1038/s41586-018-0842-3 30675058 PMC6420067

[B127] Yi W. Schlüter D. Wang X. (2019). Astrocytes in Multiple Sclerosis and Experimental Autoimmune Encephalomyelitis: Star-Shaped Cells Illuminating the Darkness of Cns Autoimmunity. Brain Behav. Immun. 80, 10–24. 10.1016/j.bbi.2019.05.029 31125711

[B128] Yin L.-L. Lin L.-L. Zhang L. Li L. (2012). Epimedium Flavonoids Ameliorate Experimental Autoimmune Encephalomyelitis in Rats by Modulating Neuroinflammatory and Neurotrophic Responses. Neuropharmacology 63 (5), 851–862. 10.1016/j.neuropharm.2012.06.025 22728315

[B129] Yin L. Chen Y. Qu Z. Zhang L. Wang Q. Zhang Q. (2014). Involvement of Jak/Stat Signaling in the Effect of Cornel Iridoid Glycoside on Experimental Autoimmune Encephalomyelitis Amelioration in Rats. J. Neuroimmunology 274 (1-2), 28–37. 10.1016/j.jneuroim.2014.06.022 25012120

[B130] Yu L. Croze E. Yamaguchi K. D. Tran T. Reder A. T. Litvak V. (2015). Induction of a Unique Isoform of theNCOA7Oxidation Resistance Gene by Interferon β-1b. J. Interferon Cytokine Res. 35 (3), 186–199. 10.1089/jir.2014.0115 25330068 PMC4350146

[B131] Zettl U. K. Hecker M. Aktas O. Wagner T. Rommer P. S. (2018). Interferon β-1a and β-1b for Patients with Multiple Sclerosis: Updates to Current Knowledge. Expert Rev. Clin. Immunol. 14 (2), 137–153. 10.1080/1744666X.2018.1426462 29318902

[B132] Zhang Y. Yin L. Zheng N. Zhang L. Liu J. Liang W. (2017). Icariin Enhances Remyelination Process After Acute Demyelination Induced by Cuprizone Exposure. Brain Res. Bull. 130, 180–187. 10.1016/j.brainresbull.2017.01.025 28161197

[B133] Zhong M. van der Walt A. Campagna M. P. Stankovich J. Butzkueven H. Jokubaitis V. (2020). The Pharmacogenetics of Rituximab: Potential Implications for Anti-cd20 Therapies in Multiple Sclerosis. Neurotherapeutics 17 (4), 1768–1784. 10.1007/s13311-020-00950-2 33058021 PMC7851267

[B134] Zhou Y. Leng X. Luo S. Su Z. Luo X. Guo H. (2016). Tolerogenic Dendritic Cells Generated with Tofacitinib Ameliorate Experimental Autoimmune Encephalomyelitis through Modulation of Th17/Treg Balance. J. Immunol. Res. 2016, 1–13. 10.1155/2016/50215372016 PMC5187469 28070525

[B135] Zhovtis Ryerson L. Li X. Goldberg J. D. Hoyt T. Christensen A. Metzger R. R. (2020). Pharmacodynamics of Natalizumab Extended Interval Dosing in Ms. Neurol. Neuroimmunol Neuroinflamm 7 (2), e672. 10.1212/NXI.0000000000000672 32019876 PMC7057061

[B136] Ziemssen T. (2011). Symptom Management in Patients with Multiple Sclerosis. J. Neurol. Sci. 311 (Suppl. 1), S48–S52. 10.1016/S0022-510X(11)70009-0 22206767

